# Gene co-expression network analysis reveals relationship between leukocyte fraction and genomic instability in dedifferentiated liposarcoma

**DOI:** 10.22099/mbrc.2025.51329.2050

**Published:** 2025

**Authors:** Mohammad Darzi, Mahdieh Shokrollahi-Barough, Elahe Nazeri, Keivan Majidzadeh-A, Rezvan Esmaeili

**Affiliations:** 1 Genetics Department, Breast Cancer Research Center, Motamed Cancer Institute, ACECR, Tehran, Iran; 2 Medical Informatics Research Group, Breast Cancer Research Center, Motamed Cancer Institute, ACECR, Tehran, Iran; 3 ATMP Department, Breast Cancer Research Center, Motamed Cancer Institute, ACECR, Tehran, Iran; 4 Department of Immunology, School of Medicine, Iran University of Medical Sciences, Tehran, 1449614535, Iran

**Keywords:** WGCNA, TIMER, DDLPS, Co-expression Network, Leukocyte Fraction, System Biology

## Abstract

Dedifferentiated Liposarcoma (DDLPS) is one of the common subtypes of liposarcoma that is considered a highly malignant category. This study aims to investigate DDLPS through a system biology approach. The gene expression profiles and clinical traits of the DDLPS were acquired from The Cancer Genome Atlas (TCGA). The identification of co-expressed modules was conducted using the weighted gene co-expression network analysis. The immune cell-related gene function was studied by a web-based tool, TIMER, and, the survival analysis was performed at both the module and single-gene levels through Cox Regression analysis. Gene enrichment analysis was also conducted using the DAVID tool. One of the nine co-expressed DDLPS modules was significantly correlated with leukocyte fraction, hyper/hypo methylation, tumor purity, and chromosome instability (CIN). Based on the biological processes used to classify genes, the hub genes in a particular module play important roles in DNA repair, microtubule organizing clusters, mitotic checkpoint dysregulation, and cell proliferation signaling pathways. After screening the genes based on intra-module connectivity, module membership, and gene significance *RAD54L* was selected as one of the important hub genes. *RAD54L* showed poor prognosis to the overall survival (OS) analysis (HR=1.6, 95% CI=1.1–2.4, p=0.02). No co-expressed modules had relationship with OS. Through DDLPS traits, CIN and hyper/hypo methylation had significant negative relationship with OS. Our achievement confirmed the inverse association between tumor purity for DDLPS gene profiles and leukocyte fraction and negative leukocyte fraction (LF) gene significance in some genes was justified according to the sub-population analyses of immune cells in TIMER.

## INTRODUCTION

Liposarcoma (LPS) is a soft tissue sarcoma that constitutes approximately 20% of all malignant mesenchymal neoplasms. LPS can be classified into four subtypes, based on their unique clinical and biological features, which include well-differentiated LPS (WDLPS)/ Atypical lipomatous tumor (ALT), dedifferentiated LPS (DDLPS), round cell/myxoid LPS (MLPS), and pleomorphic LPS. WDLPS and DDLPS, the two most prevalent subtypes of LPS, account for about 45% of all liposarcomas [1]. DDLPS is considered a highly malignant liposarcoma that develops from WDLPS that has been dedifferentiated [2]. 

Genetic studies in DDLPs are limited. Genetically, DDLPS and WDLPS are characterized by potential gene amplification of chromosome 12 (12q13-15), especially in *MDM2* and cyclin-dependent kinase 4 (*CDK4*) oncogenes, providing several roles in tumorigenesis [3, 4]. Furthermore, the overexpression of these two genes' proteins in these tumors has been reported [5]. MDM2, a negative regulator of p53, and CDK4, a crucial regulator of the G1/S cell cycle checkpoint, are amplified in 100% and 92% of patients, respectively [1, 4]. Other genes amplified on this chromosomal area are *HMGA2*, *TSPAN31*, *DDIT3*, and *FRS2*, which could be involved in the pathogenesis of DDLPS [4, 6]. The comprehensive genomic analysis explored the clinical characteristics of DDLPS with amplification of 12q13-15. Based on this investigation, the clinical prognosis could be predicted depending on somatic copy-number alterations (SCNA) and pattern of DNA methylation [1]. The clustering of DDLPS tumors into three groups, with independent clinical outcomes, is reported based on the correlation between clinical characteristics and SCNAs [3].

Unfortunately, notable local and metastatic recurrence rates are reported due to the deep location and, subsequently, incomplete surgical resection [2]. Regarding the limitations of surgery, chemotherapy, and radiotherapy in the treatment of sarcoma, there is a need to develop new treatment strategies based on the molecular heterogeneity of different subgroups of liposarcomas. A significant obstacle to the development of personalized therapies is the distinct genetic, epigenetic, and transcriptome variations in sarcomas [1].

Unrevealing the underlying disease mechanisms helps us to develop potential targeted therapies. Gene dysregulation is not the only factor that should be assessed in carcinogenesis; It is crucial to assess the gene regulatory interactions that cause heterogeneity. Since focusing only on the differential expressed genes (DEGs) limits the concurrent recognition of many other genes, potential target genes should be assessed by modeling gene interactions. Recently, patient-specific gene regulatory network analysis proposed a potential personalized medicine approach in leiomyosarcoma [7].

In this paper, weighted gene co-expression analysis (WGCNA), a framework for constructing gene co-expression networks, was applied to find the co-expressed genes related to clinicopathologic traits in liposarcoma patients. Finally, related biological pathways and significance genes were investigated. The main goal was to use the available data collection on DDLPS to study the genetic background of some genes involved in carcinogenic characteristics. The characteristics related to leukocyte fraction, hyper/hypo DNA methylation, chromosomal instability, and tumor purity in the tumor mass were important phenotypic specifications investigated in this study.

## MATERIALS AND METHODS

### The Study Design:

**Figure 1:**
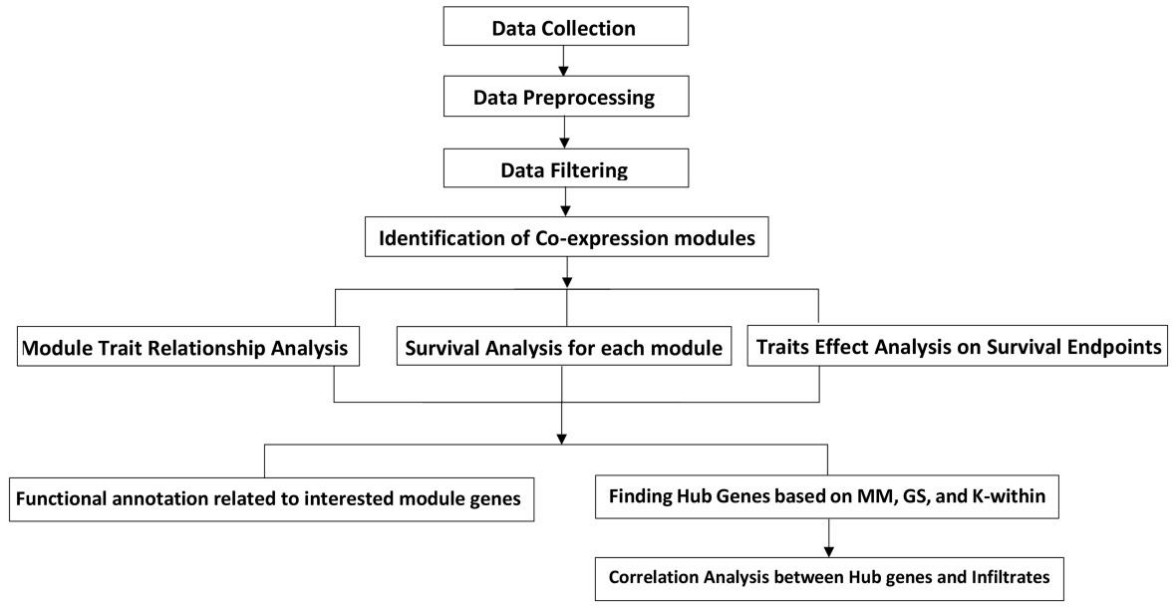
DDLPS Research Workflow Diagram. The flow diagram depicts the sequential steps for preparing and analyzing DDLPS data. Data preparation consists of the processes of "Data Collection", "Data Preprocessing", and "Data Filtering". DDLPS data were obtained from the TCGA. The next step is to identify the co-expression modules. Following the construction of the DDLPS co-expression, three analyses are carried out: the "Module Trait Relationship Analysis", the "Module Survival Analysis", and the "Traits Effect Analysis on Survival Endpoint". After that, GOs and pathways connected to each module's top ranked Gene Significance are investigated. Likewise, hub genes identified based on modules network properties such as K-within, MM, and GS.

The phases and conceptual designs of this research were illustrated in a workflow diagram based on the executed steps (Fig. 1). The first three parts of this research are data collection, preprocessing, and filtering. The DDLPS co-expression network was built in the fourth step using the provided gene expression data. The "module-trait relationship", "survival analysis for each module", and "trait effect analysis on survival endpoint" were all done individually in the following phase. Finally, in each module, gene ontologies and pathways associated with some selected genes were studied. The WGCNA gene significance assessment was used to define the basis of gene selection [8]. Each module contains the hub genes, and the computational analysis was carried out using the R platform (version 3.6.1). 

### Dataset and Preprocessing:

Dediferentied liposarcoma RNA-seq datasets were investigated on different databases. The cancer genome atlas (TCGA) as one of the main and perfect databases was used to obtain DDLPS gene expression data, overal survival, and tumor size of patients. Likewise, some clinical trait such as leukocyte fraction, tumor purity, and chromosome instability were extracted through paper entitled “Comprehensive and Integrated Genomic Characterization of Adult Soft Tissue Sarcomas”. That was written by the cancer genome atlas research network. In this paper, the authors estimated leukocyte fraction in each tumor by calculating the leukocyte signature from methylation results as described in that paper. In addition, tumor purity estimates were calculated using the ABSOLUTE algorithm [9].

**Figure 2:**
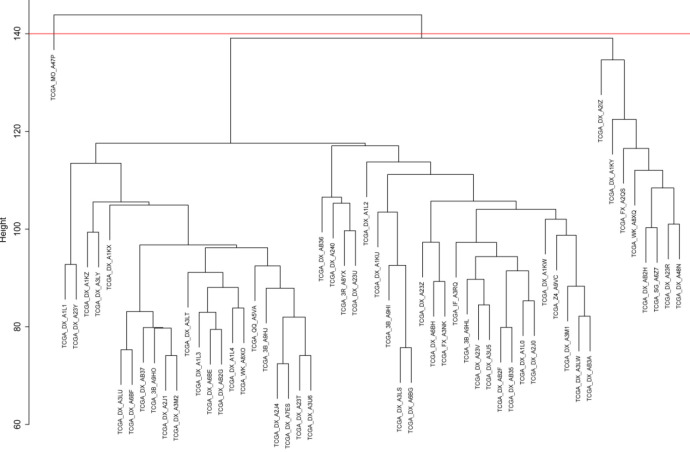
TCGA 57 DDLS via TPM Normalization Sample clustering to detect outliers

Related to finding hypo/hyper methylation trait, they applied unsupervised consensus clustering of DNA methylation on DDLPS samples. All related results for hypo/hyper meth, leukocyte fraction, tumor purity, and CIN are accessible in supplementary of that paper [1, 10]. The TCGAbiolinks package was utilized to download TCGA DDLPS count files [11]. The Transcripts Per Million (TPM) method was used to normalize the obtained TCGA expression data. The log2 function was applied to transfer normalized data to a new space. The "BatchQC" package was utilized to detect batch effects [12], and the batch effect correction was carried out using the "sva" package [13]. BatchId was defined as "PlateId", "ShipDate", and "Tissue Source Site" in relation to TCGA data. With the parametric adjustment for reducing batch effects in the TCGA dataset, the ComBat function in the "sva" package was invoked (Supplementary Fig. S1). Outlier detection was performed through hierarchical clustering on DDLPS samples during the data preprocessing step. Using the "avarage" method, the hclust function was used to create hierarchical clustering (Fig.2). To the adaptive branch pruning, the "dynamicTreeCut" package was applied to the samples' dendrogram. It was evident from the cutreeDynamic function through "tree" method (argument) that one sample was an outlier (Supplementary Fig. S2). The goodSamplesGenes function in the WGCNA package was called to remove missing entries in the DDLPS dataset.

This study focused on protein-coding genes and the analysis was limited to the most connected genes with non-zero variance. Connectivity was calculated between all protein-coding genes using the soft connectivity function (softConnectivity) from the WGCNA package [8]. Subsequently, the top 5,000 genes were chosen as the top 5000 most connected genes. 

### Weighted Gene Co-expression Network Construction:

DDLPS co-expression network was constructed through the WGCNA package. WGCNA constructs a network based on the correlation between pairs of genes. To calculate the correlation between each pair of genes, the biweight midcorrelation (bicor) method was used in this study [14]. Table S1 presents all functions involved in constructing the DDLPS co-expression network. We used the signed hybrid type for the co-expression network.

### Finding Significant Clinical Modules:

 The correlation between WGCNA-released modules and traits, and survival analysis is used to find significant clinical modules. Module eigengene (ME) was applied to calculate the relationship between modules and clinical traits. The selected traits include Age, Tumor size, Leukocyte Fraction (LF), Cancer DNA fraction, Hypo/Hyper Methylation, Purity, and Chromosome instability (CIN). The Pearson correlation coefficient was performed to identify desirable modules. Likewise, the absolute correlation value between the gene and the trait was used to calculate trait-based gene significance. Consequently, module significance (MS) was calculated by each module's average gene significance (GS) measurements, and the interested significant module–traits were selected based on a high correlation between modules and traits. 

### Hub Genes Identification:

In general, high module membership (MM) and gene significance (GS) values indicate the biological and statistical relevance of a gene. We also employed the high intra-connectivity (K-within), in accordance with the gene's MM and GS values, for hub gene discovery, per the study of Y. Lou et al. [15]. The module membership was measured with a correlation study between each gene and module eigengene. The interconnectivity was calculated by intramodularConnectivity function in WGCNA [9]. Specifically, LF analysis was performed for immune cell function/features-related genes using TIMER as a web-based tool [16].

### Survival Analysis:

 Two packages, Survival [17] and Survminer [18] were used to identify the relationship between traits and survival endpoints. The effect of tumor size, cancer DNA fraction, hypo/hyper methylation, purity, and CIN as independent variables on survival endpoints were checked through univariate Cox regression. Overall survival (OS) and progression-free interval (PFI) were studied for survival analysis. The relationship between the DDLPS co-expression network modules and the survival endpoints was investigated in relation to identifying relevant clinical modules. The ME, as the representative of each module, was selected to define the association of each module with survival endpoints. So, each ME was classified as "low" (i.e., –) and "high" (i.e., +) for multigene associations [19]. After that, univariate Cox regression, Hazard Ratio (HR), and K-M plot were executed for each module using log-rank tests. Finally, single gene survival analysis was utilized through RegParallel [20].

### Functional Annotation:

Gene enrichment analysis was conducted using the database for annotation, visualization, and integrated discovery (DAVID) [21]. Depending on the DAVID outcome, gene ontology (GO) and various pathways for selected genes were investigated in the case of biological process (BP), molecular function (MF), and cellular component (CC). To explore relevant biological pathways, we studied selected genes in the Kyoto Encyclopedia of Genes and Genomes (KEGG) and Reactome. p-value and false discovery rate (FDR) were considered <0.05 as significant ones. The selected hub genes were sub-classed into some categories in the case of molecular biology pathways and their main impact on mitosis, DNA replication, and cell proliferation-related genes using NCBI-KEGG platforms.

## RESULTS

Among fifty-eight patients with DDLPS in TCGA, two cases, including "TCGA_DX_ A7EI" and "TCGA_MO_A47P" were removed in the preprocessing step. The first one belongs to a batch with just one patient, and the second one was an outlier. The dendrogram of DDLPS samples for pruning has been shown in supplementary figure S2. The soft-thresholding approach was applied to the construction of the DDLPS co-expression network. According to the network topology analysis for different soft-thresholding powers, (β=1-20), the optimum β was considered 12 to meet the scale-free topology by fit index greater than 0.85. The detailed results of several powers for finding a network with scale-free topology properties are shown in supplementary figure S3. 

**Figure 3:**
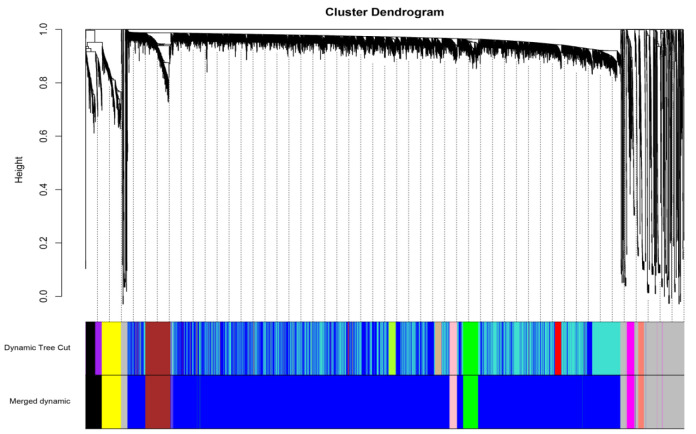
Gene dendrogram and module colors for TCGA DDLPS

The Log connectivity (k) and probability of connectivity (P(k)) were investigated through scaleFreePlot After TOM building, the hierarchical clustering was created based on the TOM dissimilarity measure, as shown in Figure 3. We identified fourteen modules through this process which decreased to nine after the merging procedure (Fig. 3). 

**Figure 4:**
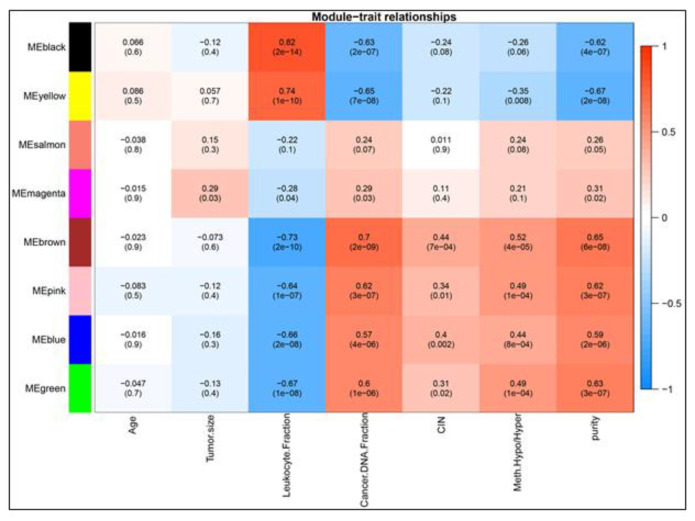
DDLPS module-trait relationship heatmap. The module-trait relationships were demonstrated by correlation values and p-values (in parenthesis) with a range of colors. The degree of correlation between modules and clinical features is also shown.

The result of this analysis is shown in Figure 4. Yellow and black modules indicated a strong positive correlation with leukocyte fraction, but brown, pink, blue, and green modules were negatively correlated. The relationship between the leukocyte fraction and the cancer DNA fraction with modules is inverse. In CIN evaluation as the next clinical feature, four modules showed a positive correlation; One module (yellow) negatively correlates with methylation and regarding this trait, four modules indicate a positive relationship (Fig. 4).

Due to the modules-traits relationship assessment, only two modules negatively correlated with the tumor purity, and the others had a positive correlation. The module significance was calculated for modules with a high correlation coefficient. According to the findings derived from the analysis of the module-traits relationship, we selected one module based on the overlap of the maximum trait number (brown), which has the highest negative correlation value for LF. black module has the highest positive correlation value in LF (Table 1). In the next step, we focused on some genes which will be defined in the hub gene identification part.

We separately ranked and selected the quarterly top genes in interested module (brown module) based on module membership (MM), intramodular connectivity, and gene significance. The common genes of these three vectors were picked as hub genes. All cumulative-intersectional analyses and data sorting results are based on high negative/positive GS values and highly positive scores of MM and K within the brown module. Obviously, hub genes were selected with emphasis on trait-specific GS values. According to our findings, 19 hub genes had high intersectional values for four traits (Table 2). These genes were classified into four sub-classes based on their functionality and impact on oncogenesis and cell proliferation pathways. Notably, these sub-classes encompass genes with significant roles in microtubule assembly (MTOC), mitosis checkpoints dysregulation, DNA repair, and DNA binding proteins (DBP) related to chromosome stability.

**Table 1:** Module-trait Correlation and module significance. The interested significant module was shown and highlighted in correlation value and module significance

**Trait**	**Module**	**Gene No.**	**Correlation Value**	**Module Significance**	**Max GS**	**Min GS**
**Leukocyte Fraction**	Black	135	0.82	0.72	0.85	0.54
	Blue	3712	-0.66	0.52	-0.82	-0.026
	Brown	210	-0.73	0.63	-0.77	-0.41
	Green	129	-0.67	0.59	-0.72	-0.45
	Pink	70	-0.64	0.55	-0.7	-0.3
	Yellow	160	0.74	0.67	0.79	0.4
**Tumor Purity**	Blue	3712	0.44	0.34	0.62	0.26
	Brown	210	0.52	0.45	0.64	0.26
	Green	129	0.49	0.43	0.59	0.26
	Pink	70	0.49	0.42	0.54	0.24
	Yellow	160	-0.35	0.32	-0.45	-0.25
**CIN**	Brown	210	0.44	0.38	0.56	0.26
**Tumor Size**	Magenta	33	0.29	-	0.37	0.25

**Table 2:** Selected hub genes in the brown module

**Gene ID**	**Subclass**	**LF-GS**	**Purity-GS**	**Methylation-GS**	**CIN-GS**	**K-within**	**MM**
***CKAP2L***	MTOC	-0.711	0.63	0.469	0.481	29.312	0.964
***KIF18B***	MTOC	-0.731	0.661	0.514	0.524	28.188	0.96
***ARHGAP11A***	Mitosis_checkpoints	-0.677	0.597	0.474	0.436	26.508	0.953
***TOP2A***	DBP	-0.693	0.637	0.498	0.453	23.725	0.953
***RAD54L***	DBP	-0.665	0.59	0.473	0.414	22.452	0.943
***EXO1***	DNA_repair	-0.723	0.594	0.513	0.444	20.789	0.94
***ESPL1***	MTOC	-0.752	0.7	0.467	0.444	20.609	0.938
***BUB1***	MTOC	-0.67	0.648	0.495	0.498	21.748	0.934
***MKI67***	Mitosis_checkpoints	-0.722	0.685	0.489	0.48	20.871	0.926
***KIF15***	MTOC	-0.702	0.653	0.495	0.406	14.174	0.924
***KIF4A***	MTOC	-0.677	0.616	0.478	0.493	19.816	0.923
***PRC1***	MTOC	-0.702	0.673	0.464	0.448	20.236	0.923
***SPAG5***	MTOC	-0.718	0.675	0.472	0.435	17.93	0.918
***TICRR***	Mitosis_checkpoints	-0.746	0.63	0.519	0.44	15.118	0.916
***CEP85***	MTOC	-0.71	0.646	0.525	0.413	12.815	0.916
***CEP78***	MTOC	-0.752	0.633	0.595	0.489	14.362	0.913
***ESCO2***	DBP	-0.665	0.619	0.49	0.406	14.197	0.906
***SMC4***	DBP	-0.642	0.577	0.463	0.39	10.748	0.88
***SFPQ***	DBP	-0.718	0.665	0.466	0.412	8.442	0.865

All continued traits were dichotomized for survival analysis according to the median values. CIN and hyper/hypo methylation had a significant relationship with the overall survival of DDLPS patients. The hazard ratio amounts show a higher value for the hypermethylated group in favor of the mortality risk (Table 3). 

**Table 3:** Univariate survival analysis was considered for DDLSP traits with OS, and PFI as endpoints.

	**Median**		** OS**					** PFI**		
		**beta**	**HR**	**p-value**	**CI**		**beta**	**HR**	**p-value**	**CI**
**Leukocyte Fraction**	0.27	-0.25	0.781	0.595	0.31-1.94		0.46	1.57	0.26	0.7-3.48
**CIN **	406	1.14	3.13	0.03	1.14-8.56		0.32	1.38	0.41	0.64-3.00
**Methylation**	-	1.56	4.8	0.002	1.74-13.08		0.21	1.24	0.634	0.51-2.97
**Purity**	0.69	-0.17	0.84	0.721	0.32-2.18		-0.65	0.52	0.13	0.22-1.21

The results of OS and PFI analyses show that DDLPS patients with a higher CIN will have a bad prognosis and a higher risk of death. Also, the survival curves plotted through Kaplan-Meier show poor OS for methylation, and CIN (Supplementary Fig. S4).

This study's univariate and multivariate analysis detected no modules significantly associated with survival endpoints (Supplementary Table S2). Likewise, the single gene survival analysis was performed on all genes without module partitioning. In 

Table 4, the results of univariate survival analysis on 19 hub genes were presented. For *RAD54L*, *CEP78*, and *SFPQ*, the survival analysis p-value was less than 0.05. It revealed that, out of the 19 hub genes, they were prognostic genes. Three prognostic genes have a hazard ratio greater than 1. This indicates that *SFPQ*, *CEP78*, and *RAD54L* were poor prognostic genes.

**Table 4:** Single gene overall survival analysis on hub genes

**No**	**Gene ID**	**p-Value**	**Hazad Ratio**	**HRlower**	**HRupper**
**1**	***CKAP2L***	0.10	1.38	0.94	2.03
**2**	***KIF18B***	0.07	1.42	0.96	2.09
**3**	***ARHGAP11A***	0.13	1.34	0.91	1.96
**4**	***TOP2A***	0.06	1.45	0.97	2.15
**5**	***RAD54L***	0.03	1.56	1.05	2.30
**6**	***EXO1***	0.11	1.36	0.93	1.98
**7**	***ESPL1***	0.07	1.42	0.97	2.07
**8**	***BUB1***	0.13	1.32	0.91	1.92
**9**	***MKI67***	0.21	1.29	0.86	1.92
**10**	***KIF15***	0.08	1.40	0.95	2.05
**11**	***KIF4A***	0.24	1.25	0.86	1.83
**12**	***PRC1***	0.15	1.33	0.89	1.98
**13**	***SPAG5***	0.45	1.16	0.78	1.72
**14**	***TICRR***	0.13	1.34	0.91	1.97
**15**	***CEP85***	0.09	1.39	0.94	2.05
**16**	***CEP78***	0.04	1.51	1.01	2.25
**17**	***ESCO2***	0.32	1.20	0.83	1.74
**18**	***SMC4***	0.10	1.39	0.94	2.07
**19**	***SFPQ***	0.04	1.52	1.02	2.26

All significant modules with a high module–trait correlation was analyzed. To find biological processes and pathways, we examined the top genes with the highest level of gene significance value in each module. The black module genes were significantly enriched for immune response-related genes such as T-cell costimulation, and leukocyte migration (Table 5). 

These genes were also found in Antigen processing and presentation, rheumatoid arthritis pathogenicity, tuberculosis-related immunity, and cell adhesion molecules (CAMs) related pathways. Vice versa, genes in the brown module related to leukocyte fraction were independent of the immune response function. These belong to DNA repair, cell division, mitotic sister chromatid segregation, sister chromatid cohesion, and DNA replication and these achievements can justify the negative correlation value brown module. We used TIMER to do a detailed analysis of LF-related genes in the brown module, and its result except for a few genes confirmed our findings (Supplementary Table S3). These genes had positive correlation values in brown module which were negative in our data, detailed analyses showed these belong to immunosuppressive immune cells such as regulatory T cells and myeloid-derived suppressor cell (Supplementary Fig. S5). 

**Table 5:** Functional annotation terms in the relative significance module with leukocyte fraction

**Functional Annotation Term**	**Count**	**FDR**		**Functional Annotation Term**	**Count**	**FDR**
**Black module**						
**GO-Biological Process (BP)**				** KEGG Pathway**		
immune response	9	1.68E-05		Antigen processing and presentation	5	2.43E-04
T cell costimulation	5	2.61E-04		Rheumatoid arthritis	5	2.43E-04
Leukocyte migration	4	0.017		Tuberculosis	5	4.30E-04
**GO-Molecular Function (MF)** Cell adhesion molecules (CAMs) 5 0.053052						
MHC class II receptor activity	4	4.28E-05		Herpes simplex infection5	5	6.70E-04
MHC class II protein complex binding	4	4.28E-05		**REACTOME Pathway**		
**GO-Cellular Component (CC)** **MHC class II antigen presentation **5 3.40E-05						
Plasma membrane	17	6.87E-04		Translocation of ZAP-70 to Immunological synapse	4	1.81E-05
An integral component of the membrane	17	0.005		Phosphorylation of CD3 and TCR zeta chains	4	1.81E-05

**Brown module**						
**GO-Biological Process (BP)**				**GO-Cellular Component (CC)**		
DNA repair	6	0.003		Cytoplasm	19	0.004
cell division	6	0.011		Nucleus	19	0.004
Mitotic sister chromatid segregation	4	0.001		Nucleoplasm	12	0.019
Sister chromatid cohesion	4	0.024		Centrosome	6	0.008
DNA replication	4	0.052				

The gene ontology analyses related to hyper/hypo methylation in the brown module showed that these genes were enriched for DNA replication, DNA repair, DNA-protein, and ATP binding pathways. Also, genes related to cellular components in that module were highlighted for nucleoplasm and nucleus. As provided in Table 6, based on the KEGG pathway database, genes would be classified in cell cycle and DNA replication pathways and according to the Reactome analyses, DNA replication-related regulatory pathways were found for significant genes of this module.

**Table 6:** Functional annotation terms in brown module with methylation

**Functional Annotation Term**	**Count**	**FDR**	**Functional Annotation Term**	**Count**	**FDR**
**GO-Biological Process (BP)**			**GO-Cellular Component (CC)**		
DNA replication	12	1.51E-13	Nucleoplasm	19	6.53E-07
DNA repair	9	3.83E-07	Nucleus	18	0.019
Strand displacement	4	6.88E-04	**KEGG Pathway**		
DNA replication initiation	4	9.77E-04	Cell cycle	5	2.13E-04
DNA synthesis involved in DNA repair	4	0.001	DNA replication	3	0.005
**GO-Molecular Function (MF)**			**REACTOME Pathway**		
Protein binding	23	0.003	Activation of ATR in response to replication stress	5	6.33E-05
DNA binding	14	8.09E-06	Assembly of the pre-replicative complex	4	1.07E-04
ATP binding	12	1.00E-04	Homologous DNA Pairing and Strand Exchange	4	2.42E-04

Among six significant modules related to tumor purity, we concentrate on the brown module as the most correlated module with tumor purity. As illustrated in Table 7, genes in the brown module were enriched cell division, mitotic nuclear division, microtubule-based movement, sister chromatid cohesion, and cell proliferation.

**Table 7:** Functional annotation terms in brown modules with purity

**Functional Annotation Term**	**Count**	**FDR**	**Functional Annotation Term**	**Count**	**FDR**
**GO-Biological Process (BP)**			**GO-Cellular Component (CC**)		
Cell division	12	2.27E-10	Nucleus	19	3.07E-04
Mitotic nuclear division	10	5.12E-09	Cytoplasm	17	0.002
Microtubule-based movement	6	4.94E-06	Cytosol	14	0.001
Sister chromatid cohesion	6	1.32E-05	Nucleoplasm	13	0.001
Cell proliferation	6	0.005	KEGG Pathway		
Mitotic metaphase plate congression	3	0.02	Cell cycle	4	0.002
**GO-Molecular Function (MF)**			Oocyte meiosis	3	0.02
Protein binding	23	0.002	REACTOME Pathway		
Microtubule binding	8	8.07E-07	Kinesins	6	6.81E-06
Microtubule motor activity	6	2.70E-06	Resolution of Sister Chromatid Cohesion	6	8.93E-05

## DISCUSSION

In this study, a system biology approach was applied to investigate the mRNA expression of DDLPS patients with the WGCNA framework. In the DDLPS co-expression network, we identified modules and genes related to clinical data such as LF, tumor purity, CIN, and hyper/hypo methylation.

5000 of all protein-coding genes were chosen for this study based on the degree of connection. eight traits-related modules were constructed and identified using the WGCNA framework. The most important genes were sorted based on GS, MM, and K-within in selected module. Two modules (brown and black) were selected because they had significant relationship with traits such as LF, CIN, Tumor purity and hyper/hypo methylations.

Here, we reviewed some of the most significant genes in the brown module as selected ones. Our findings indicate that around 10 out of the 19 hub genes in this module are crucial for microtubule polymerization, as well as for the proteins associated with the microtubule-organizing center (MTOC) that are involved in the cell cycle and mitosis. The brown module showed a negative correlation between certain genes, especially in the MTOC-related subclass, and the leukocyte fraction. This correlation is associated with high tumor purity levels, CINs, and hyper/hypo methylation. We found this paradoxical feature (negative correlation of LF with positive GSs in other traits) can be justified by the focus of these genes on immune cells' function and/or their impact on tumor immunology [22, 23]. Nevertheless, various studies have demonstrated an inverse relationship between LF and tumor purity, a finding that we corroborated in our study, especially within the black and brown modules [24]. 

An important factor in treatment is the tumor cell purity index, which can be high when the immune system function is low. However, some evidence indicates that a high tumor cell purity is associated with the absence of functional immune cells in the tumor. Reducing the tumor cell purity index in line with LF increment would be a reliable prognostic factor [25, 26]. LF trait definition is related to tumor-infiltrating lymphocytes (TILs) as markers of the immune system's function in tumors. TILs usually become residents of the tumor from mass generation or attract into the tumor due to the release of inflammatory cytokines from the tumor environment [27]. The efficient anti-tumor properties of TILs are performed through cytotoxic T cells that express CD8 and natural killer cells. Due to the tumor burden increase, tumor cells altered immune responses to favor immunosuppression, increasing T-reg cells and myeloid-derived suppressor cells (MDSCs). The low tumor purity might be related to cancer stem cell frequency and regulatory immune cell infiltration, such as M2 and MDSC cells in tumor masses [28]. The nature of TILs in the tumor site is more crucial than their frequency. It is imperative to note that cells with immunomodulatory properties can work in favor of cancer [29]. 

Some of the controversies surrounding tumor purity and LF correlation in WGCNA analysis, as well as other immune-related databases like TIMER, can be quite challenging to understand. This may be due to the dysfunction of immune cells in cancer, as well as the overexpression of inhibitory immune checkpoints in tumor regions, which can alter the immune cells' function or attract immunosuppressive cells like MDSC cells from the bone marrow [30]. Various studies have shown that these approaches can explain the positive relationship between LF and poor prognosis [31]. Overall based on our achievements the LF and tumor cell purity opposite relationship is confirmed using Estimation of STromal and Immune cells in MAlignant Tumor tissues using Expression data (ESTIMATE) scores in some malignancies, especially in melanoma [32]. 

In our achievements, the GS score of LF in many genes was negative but in complementary analyses by TIMER, we found positive GS that was completely different from our results. We need to deep into details to find the reason. The subpopulations-related correlation analysis in TIMER showed significant differences; all cells’ correlation with candidate genes was negative, but there was a robust positive correlation in some immune cells with immunosuppressive function that a high amount of correlation could compensate all negative GSs in other subpopulations. The most impressive cell in these analyses was MDSC. We found that all results of MDSCs were highly positive, which can hide other gene correlations that can switch the total negative GS of LF to positive. The correlation between positive MDSC frequency and low overall survival confirms the controversy of TIMER results and supports our accurate analysis in predicting poor prognosis for candidate genes [33].

In addition to the immune cell-related parameters and tumor cell purity, molecular and genetic parameters are also rational in determining the status of genes in personalized medicine [34]. Cell proliferation and oncogenes overexpression can be found as a result of structural alterations in chromosomes caused by any mutation or epigenetic dysregulation. This is a direct consequence of CIN features, which we have confirmed through our research [35, 36]. We decided to focus on *RAD54L* as one of these genes despite the majority of the hub genes having great scores in terms of GS and other criteria. This choice was taken regarding the high values for the WGCNA-related parameters as well as the large negative score for LF, which was entirely positive in the TIMER analysis and was determined to be positive due to MDSC cells. The articles' confirmation that higher *RAD54L* expression can cause MDSCs to be recruited to the tumor location, was another factor in the choice of this gene [37]. In the normal state of immune cells, this gene helps to regulate hypermutation (and antibody class switching but its role is not critical in this area), binds to the DNA molecule, and increases the possibility of homologous recombination (HR) by creating a break in the DNA molecule [37, 38].

RAD54L is a protein that is functionally classified in the family of DNA-binding proteins, which has a special role in DNA repair by HR. This protein helps to repair DNA breaks in somatic cells and is necessary for somatic hypermutation in immune cells to create variable regions of antibody and T cell receptors. Therefore, according to the estimates, this gene plays the same role both in immune cells as a hypermutation indicator and in other proliferating cells as a tumor suppressor gene [39]. The overexpression of *RAD54L* in tumor-surrounding lymphatic tissues, especially tertiary lymph nodes, may indicate an adaptive immune response against tumor cells, but it can also be justified as a mutation-related gene in cancer cells [40, 41]. According to our results, the relationship between *RAD54L* and the lymphocyte fraction was negative, which denies the hypermutation indicator role of *RAD54L* overexpression in the tumor. 

Experimental data on cell lines has revealed complex information about the role of this gene in HR in sarcoma. However, its overexpression in various malignancies has been reported to have a positive impact on hyper/hypo DNA methylation and DNA repair [42, 43]. *RAD54L* expression level has been altered in many types of malignancies [44, 45]. Considering the function of the *RAD54L* gene, the role of PARP inhibitors in regulating the mRNA level of this gene can be highlighted. Studies have shown that using Olaparib can significantly increase progression-free survival (PFS) in patients with RAD54L expression level change. These findings show the importance of studying the *RAD54L* in justifying the necessity of Olaparib administration in patients with metastatic cancers [46]. *RAD54L* exhibits elevated expression in the majority of tumors and is strongly associated with unfavorable survival outcomes. This gene demonstrates robust correlations with the infiltration levels of diverse immune cells, including MDSCs and other types of cells such as cancer-associated fibroblasts (CAFs), endothelial cells. Additionally, RAD54L shows strong associations with critical factors such as tumor mutation burden (TMB), microsatellite instability (MSI), multiple immune checkpoints, and immune cell infiltration. Given that RAD54L functions as a DNA-binding protein capable of accelerating mutation occurrence, targeted inhibitors specifically directed at its DNA binding are needed [47].

Understanding the role of the RAD54L gene is pivotal in elucidating the mechanism of action of PARP inhibitors. These inhibitors function by trapping poly (ADP-ribose) polymerase (PARP) on DNA, creating a physical barrier to the replication machinery. In the context of homologous recombination repair (HRR) deficiency in cancer cells, HRR is required to prevent replication forks' collapse and subsequent cell death caused by trapped PARP [48].

Highlighting the impact of PARP inhibitors on the mRNA level of *RAD54L*, studies have demonstrated that the use of Olaparib significantly enhances progression-free survival (PFS) in patients with altered *RAD54L* expression levels. This suggests a novel approach to targeted therapy in sarcoma patients. These findings underscore the importance of investigating *RAD54L* to justify the necessity of administering Olaparib in patients with metastatic cancers [49]. Due to a study, the HR score of *RAD54L* was very high [50]. It showed sensitive properties for PARP inhibitors like Olaparib, Nivaparib, and Adavosertib prescription in soft tissue sarcoma patients. Their study evaluated all soft tissue malignancies but there was no more information about DDLPS. In their study, *RAD51* loss of function was more significant in bonemarrow sarcoma but gene expression alteration was reported about *RAD54L*. 

Our achievements confirmed that due to the significant impact of *RAD54L* in DDLPS prognosis. So PARP inhibitor prescription in DDLPS would be suggested in the future [50]. The increased expression of this gene compared to other members of the RAD family such as *RAD51* is a risk factor because this protein, in case of increased expression, causes genome instability and increases the mutation load. Although this mechanism is vital in immune cells, in other somatic cells, it leads to an increase in the transformation of the nucleus of the cells into a cancer cell, and therefore, the increase in its expression as an oncogene has a bad prognosis. Studies have shown that the use of PARP inhibitors has a therapeutic role in causing more mutations because they can limit the amount of RAD54L binding to the DNA molecule [51]. As can be guessed, the very important role of RAD54L in genome instability can also justify CIN and affect the epigenomic changes related to DNA methylation [52]. Increasing the expression of this gene causes the number of DNA repair forks in the genome to exceed the manageable number, and instead of being properly repaired by homologous recombination, the genome faces more breaks and becomes cancerous [50].

According to the CINSARC signature in the sarcoma study, some important genes related to genomic instability can impair normal cell proliferation and cause continuous mutations [53]. *RAD54L* was also the prognostic gene, and its loss of function significantly increased the risk of malignancy. This study investigated the effect of LF, tumor purity, CIN, and hyper/hypo methylation on OS. Among them, hyper/hypo methylation and CIN hurt the OS of DDLPS patients. The poor impact of CIN on OS was approved previously in related studies [54, 55]. 

In conclusion, Different genes are related to LF, purity, CIN, and hyper/hypo methylation in significant modules, according to a DDLPS in silico study that utilized WGCNA as a co-expression network framework. Notably, MTOC-related genes emerged as having a critical role in oncogenesis independent of LF. Based on our findings after TIMER analysis, we can confidently assert that there is a strong correlation between LF and DDLPS-related genes. While complications in rare genes were observed, particularly concerning immune cell sub-populations, the total infiltration of immune cells did not show critical differences in various analyses. Gene screening, incorporating measurements such as K-within, GS, MM, and OS analyses, highlighted *RAD54L* as a valuable hub gene. Our achievements indicate the importance of this gene on the DDLPS overall survival, which was confirmed by the effects of DNA repair on DDLPS treatment. Conclusively, the study's findings recommend adding PARP inhibitors to the main treatment line of DDLPS chemotherapy guidelines, as this approach holds promise for enhancing overall patient survival. However, further experimental and clinical investigations are recommended to validate these findings.
