# Supplementary material for: Gene co-expression network analysis reveals relationship between leukocyte fraction and genomic instability in dedifferentiated liposarcoma
Source: Mol Biol Res Commun. 2025;14(3):203–18. doi: 10.22099/mbrc.2025.51329.2050 (PMC12046367; doi:10.22099/mbrc.2025.51329.2050)
Supplement: Supplementary file 1 — Tables S1-S3 [file MBRC-14-203-s001.pdf]

Table S1: Functions used in DDLPS Co-expression Network Construction

No.	Function name	Assigned parameters	Contribution to DDLPS co-expression construction
1	pickSoftThreshold	datExpr_DDLPS, network type = "signed hybrid", corOptions=list (use="p"), corFnc="bicor", corOptions=list(maxPOutliers =0.1), verbose = 5	calculate a set of values for soft power (β).
2	adjacency	datExpr = datExpr_DDLPS, network type = "signed hybrid", power = β, corOptions=list (use="p"), corFnc="bicor", corOptions=list(maxPOutliers =0.1)	This function converted the correlation matrix to the adjacency matrix with soft power (β).
3	TOMsimilarity	Topological Overlap Matrix (TOM) that output of adjacency function	This function calculates the similarities based on the number of shared neighbors between gene pairs in the resulting co-expression network[9].
4	hclust	Distance matrix of (1-TOM), Method = "average"	This function was utilized to run the average linkage hierarchical clustering algorithm on the (1-TOM) matrix.

No.	Function name	Assigned parameters	Contribution to DDLPS co-expression construction
5	cutreeDynamic	dendro = geneTree_DDLPS, method = "hybrid", distM = dissTOM, deepSplit = 4, pamRespectsDendro = FALSE, minClusterSize = 40	By cutreeDynamic, gene modules were identified [9].
6	labels2colors	dynamicMods	This function converts module labels to module colors.
7	moduleEigengenes	datExpr_DDLPS , colors = dynamicColors	moduleEigengenes produces a representative for each module[9]
8	mergeCloseModules	datExpr_DDLPS , dynamicColors, cutHeight = 0.09, verbose = 3	Close modules were merged by mergeCloseModules based on cutHeight.

Table S2: Survival analysis detected no modules significantly associated with survival endpoints (OS and PFI)

Module Name	No. Genes	OS			PFI		
		HR	P-value	CI	HR	P-value	CI
MEbrown	210	1.3615	0.107	0.94-1.98	0.8886	0.494	0.63-1.25
MEsalmon	50	0.91542	0.874	0.31-2.72	0.698	0.0612	0.45-1.02
MEblue	3712	1.4158	0.0867	0.95-2.11	1.2987	0.133	0.92-1.83
MEgreen	129	1.2032	0.357	0.81-1.78	0.7368	0.0776	0.53-1.03
MEblack	135	0.92114	0.699	0.61-1.39	0.7943	0.38	0.47-1.33
Mepink	70	1.4443	0.085	0.95-2.19	1.06891	0.692	0.77-1.49
MEyellow	160	0.721	0.13	0.472-1.1	1.1466	0.301	0.89-1.49
Magenta	66	1.2866	0.0903	0.96-1.72	1.686	0.1	0.76-1.62

Table S3: The correlation of some LF-Related genes in brown module with infiltrates for Sarcoma through TIMER2.0

RAD54L			
cancer	infiltrates	rho	p
SARC (n=260)	Cancer associated fibroblast_XCELL	-0.321815151	2.77E-07
SARC (n=260)	Macrophage M2_QUANTISEQ	-0.165292028	0.009695581
SARC (n=260)	MDSC_TIDE	0.42078765	6.87E-12
SARC (n=260)	Myeloid dendritic cell_TIMER	0.179307008	0.004965151
SARC (n=260)	Neutrophil_QUANTISEQ	0.190695511	0.002781038
SARC (n=260)	T cell CD4+ (non-regulatory) _QUANTISEQ	-0.184935336	0.003743598
SARC (n=260)	T cell CD4+ central memory_XCELL	-0.252793392	6.51E-05
SARC (n=260)	T cell CD4+ effector memory_XCELL	-0.188284073	0.003152732
SARC (n=260)	T cell CD4+ memory resting_CIBERSORT	-0.239734958	0.000156305
SARC (n=260)	T cell CD4+ memory resting_CIBERSORT-ABS	-0.262735655	3.24E-05
SARC (n=260)	T cell CD4+ Th2_XCELL	0.645385764	3.86E-30
SARC (n=260)	T cell CD4+_TIMER	-0.199713212	0.001717102
SARC (n=260)	T cell CD8+ naive_XCELL	0.211905807	0.000865391
SARC (n=260)	T cell CD8+_TIMER	0.213771754	0.000776578
SARC (n=260)	T cell regulatory (Tregs)_XCELL	-0.219753429	0.000545434
SARC (n=260)	Purity	0.218115927	0.000585849
SARC (n=260)	B Cell	0.146127221	0.023860399
SARC (n=260)	CD8+ T Cell	0.043692214	0.500524456
SARC (n=260)	CD4+ T Cell	-0.104820571	0.10599773
SARC (n=260)	Macrophage	-0.055831592	0.394223451
SARC (n=260)	Neutrophil	-0.067020537	0.299100305
SARC (n=260)	Dendritic Cell	0.149603568	0.020152944
CKAP2L			
SARC (n=260)	Cancer associated fibroblast_EPIC	0.153272857	0.016571508
SARC (n=260)	Cancer associated fibroblast_XCELL	-0.20133871	0.001570681
SARC (n=260)	Macrophage M2_QUANTISEQ	-0.212769082	0.000823199
SARC (n=260)	Macrophage M2_TIDE	-0.153736915	0.016242472

SARC (n=260)	Neutrophil_MCPCOUNTER	0.207655515	0.001103704
SARC (n=260)	Neutrophil_QUANTISEQ	0.170203263	0.007710819
SARC (n=260)	T cell CD4+ central memory_XCELL	-0.241282948	0.000141238
SARC (n=260)	T cell CD4+ effector memory_XCELL	-0.294261705	2.91E-06
SARC (n=260)	T cell CD4+ memory_XCELL	0.204292533	0.001333495
SARC (n=260)	T cell CD4+ Th2_XCELL	0.591885046	1.86E-24
SARC (n=260)	T cell CD4+_TIMER	-0.226233082	0.000368012
SARC (n=260)	T cell CD8+_TIMER	0.164068936	0.010255373
SARC (n=260)	Purity	0.270549332	1.76E-05
SARC (n=260)	B Cell	0.110478597	0.088339256
SARC (n=260)	CD8+ T Cell	0.130921161	0.042727615
SARC (n=260)	CD4+ T Cell	-0.213607158	0.000889227
SARC (n=260)	Macrophage	-0.118247739	0.07039028
SARC (n=260)	Neutrophil	0.03500888	0.587848298
SARC (n=260)	Dendritic Cell	0.01428899	0.825339466
KLIF18B			
SARC (n=260)	B cell_EPIC	-0.17835	0.005204
SARC (n=260)	Cancer associated fibroblast_EPIC	0.236151	0.000197
SARC (n=260)	Cancer associated fibroblast_XCELL	-0.27058	1.83E-05
SARC (n=260)	Macrophage M0_CIBERSORT	0.193286	0.002426
SARC (n=260)	Macrophage M0_CIBERSORT-ABS	0.199	0.001785
SARC (n=260)	Macrophage M2_QUANTISEQ	-0.17817	0.005252
SARC (n=260)	Macrophage M2_XCELL	-0.16491	0.009866
SARC (n=260)	MDSC_TIDE	0.508698	1.83E-17
SARC (n=260)	T cell CD4+ central memory_XCELL	-0.23361	0.000232
SARC (n=260)	T cell CD4+ effector memory_XCELL	-0.31501	5.07E-07
SARC (n=260)	T cell CD4+ memory resting_CIBERSORT	-0.18	0.004797
SARC (n=260)	T cell CD4+ memory resting_CIBERSORT-ABS	-0.2255	0.000385
SARC (n=260)	T cell CD4+ memory_XCELL	0.171426	0.007277
SARC (n=260)	T cell CD4+ Th2_XCELL	0.594148	1.12E-24

SARC (n=260)	T cell CD4+_TIMER	-0.19653	0.002041
SARC (n=260)	T cell CD8+ naive_XCELL	0.195342	0.002175
SARC (n=260)	T cell CD8+_TIMER	0.197081	0.001981
SARC (n=260)	T cell regulatory (Tregs)_XCELL	-0.17608	0.005817
SARC (n=260)	Purity	0.292149	3.30E-06
SARC (n=260)	B Cell	0.125957	0.051801
SARC (n=260)	CD8+ T Cell	0.020631	0.750506
SARC (n=260)	CD4+ T Cell	-0.15509	0.016411
SARC (n=260)	Macrophage	-0.11083	0.090036
SARC (n=260)	Neutrophil	0.010159	0.875066
SARC (n=260)	Dendritic Cell	0.018332	0.777079
CEP78			
SARC (n=260)	B cell plasma_CIBERSORT	0.159543807	0.012582906
SARC (n=260)	B cell plasma_CIBERSORT-ABS	0.162078537	0.011227605
SARC (n=260)	B cell_MCPCOUNTER	-0.152920424	0.01682527
SARC (n=260)	Cancer associated fibroblast_EPIC	0.180766233	0.00461811
SARC (n=260)	Cancer associated fibroblast_XCELL	-0.182905794	0.00414867
SARC (n=260)	Macrophage M1_XCELL	-0.208891093	0.001028854
SARC (n=260)	Macrophage M2_QUANTISEQ	-0.290353543	3.99E-06
SARC (n=260)	Macrophage M2_XCELL	-0.225954318	0.000374382
SARC (n=260)	Macrophage_EPIC	-0.160476522	0.012068309
SARC (n=260)	Macrophage_TIMER	-0.200303704	0.001662527
SARC (n=260)	Macrophage_XCELL	-0.196570005	0.002036154
SARC (n=260)	Macrophage/Monocyte_MCPCOUNTER	-0.228408972	0.00032165
SARC (n=260)	MDSC_TIDE	0.503303064	4.49E-17
SARC (n=260)	Myeloid dendritic cell_XCELL	-0.284857188	6.16E-06
SARC (n=260)	Neutrophil_QUANTISEQ	0.155259957	0.01520233
SARC (n=260)	NK cell_EPIC	-0.215632911	0.000696438
SARC (n=260)	Plasmacytoid dendritic cell_XCELL	-0.167752459	0.008650833
SARC (n=260)	T cell CD4+ effector memory_XCELL	-0.3092647	8.33E-07

SARC (n=260)	T cell CD4+ memory_XCELL	0.17004657	0.007768078
SARC (n=260)	T cell CD4+ Th1_XCELL	-0.180275491	0.004732306
SARC (n=260)	T cell CD4+ Th2_XCELL	0.432418204	1.54E-12
SARC (n=260)	T cell CD4+_TIMER	-0.330009383	1.31E-07
SARC (n=260)	T cell CD8+ central memory_XCELL	-0.164935284	0.009855992
SARC (n=260)	T cell CD8+ naive_XCELL	0.186378958	0.003477591
SARC (n=260)	T cell regulatory (Tregs)_XCELL	-0.188936778	0.003047921
ESPL1			
SARC (n=260)	Cancer associated fibroblast_EPIC	0.230491	0.000282
SARC (n=260)	Cancer associated fibroblast_XCELL	-0.25085	7.44E-05
SARC (n=260)	Macrophage M0_CIBERSORT	0.168563	0.008329
SARC (n=260)	Macrophage M0_CIBERSORT-ABS	0.175568	0.005964
SARC (n=260)	Macrophage M2_QUANTISEQ	-0.16427	0.010161
SARC (n=260)	Macrophage M2_XCELL	-0.24315	0.000125
SARC (n=260)	MDSC_TIDE	0.501409	6.13E-17
SARC (n=260)	Myeloid dendritic cell_QUANTISEQ	0.180914	0.004584
SARC (n=260)	Neutrophil_MCPCOUNTER	0.158336	0.013278
SARC (n=260)	T cell CD4+ effector memory_XCELL	-0.28028	8.79E-06
SARC (n=260)	T cell CD4+ memory resting_CIBERSORT-ABS	-0.1886	0.003101
SARC (n=260)	T cell CD4+ Th2_XCELL	0.496153	1.44E-16
SARC (n=260)	T cell CD4+_TIMER	-0.16471	0.009957
SARC (n=260)	T cell CD8+ naive_XCELL	0.179254	0.004978
SARC (n=260)	T cell regulatory (Tregs)_XCELL	-0.16894	0.008184
SARC (n=260)	Purity	0.278029	1.00E-05
SARC (n=260)	B Cell	0.074482	0.251375
SARC (n=260)	CD8+ T Cell	-0.02067	0.750097
SARC (n=260)	CD4+ T Cell	-0.13705	0.034205
SARC (n=260)	Macrophage	-0.13371	0.040565
SARC (n=260)	Neutrophil	0.077908	0.227233
SARC (n=260)	Dendritic Cell	-0.06596	0.307836
