# Supplementary material for: Gene co-expression network analysis reveals relationship between leukocyte fraction and genomic instability in dedifferentiated liposarcoma
Source: Mol Biol Res Commun. 2025;14(3):203–18. doi: 10.22099/mbrc.2025.51329.2050 (PMC12046367; doi:10.22099/mbrc.2025.51329.2050)
Supplement: Supplementary file 2 — Figures S1-S5 [file MBRC-14-203-s002.pdf]

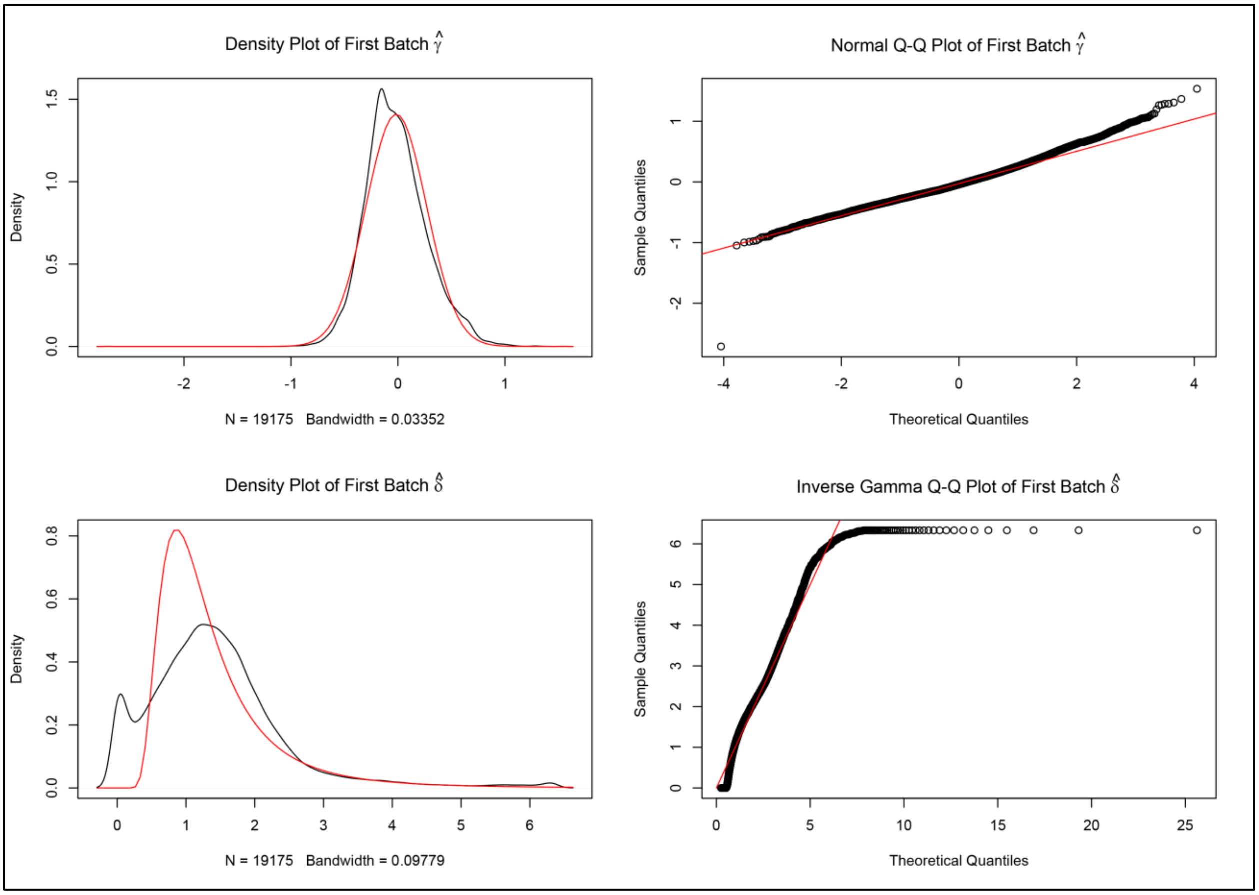

Figure S1: The R plot which generated by running Combat function on 58 TCGA DDLPS samples

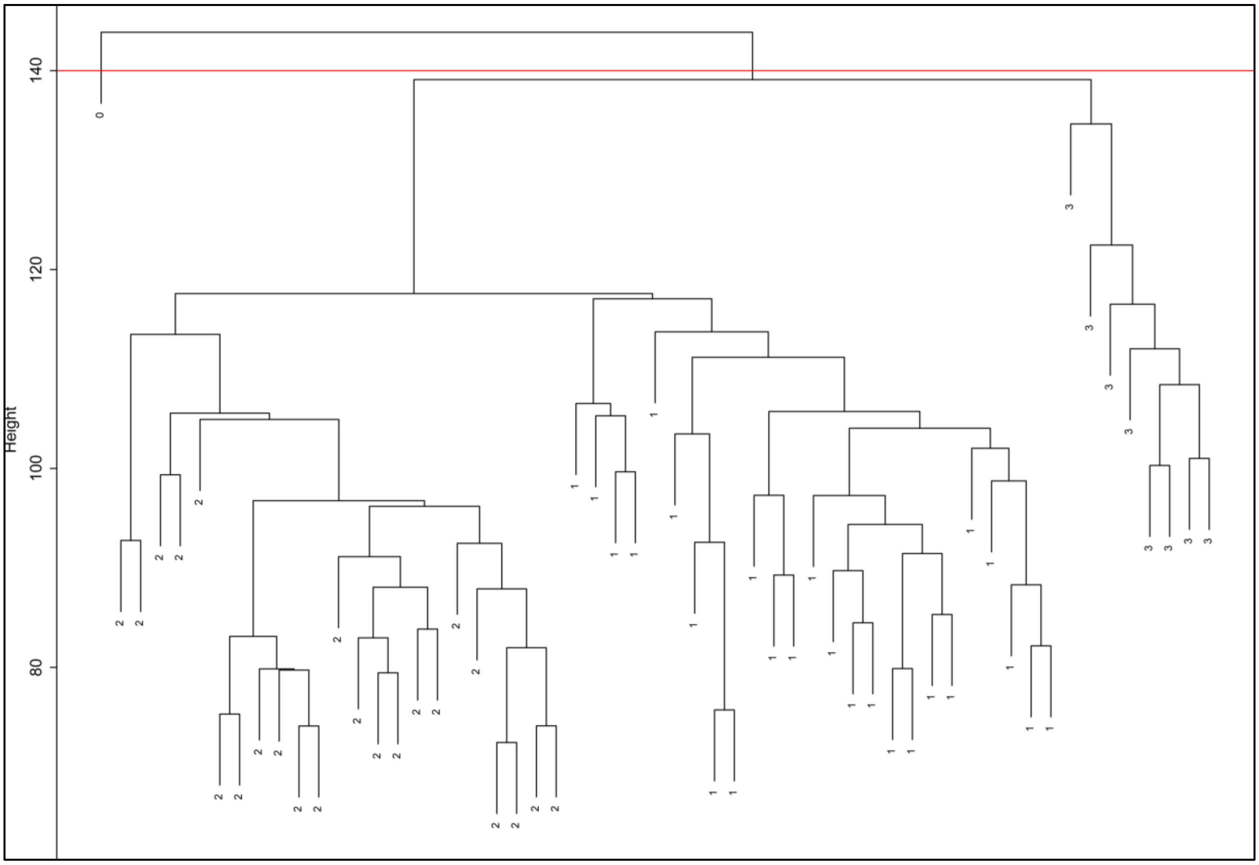

Figure S2: The R plot for hierarchical clustering on 58 TCGA DDLPS Samples to detect outliers

DDLPS samples were utilized to find and eliminate outliers using hierarchical clustering. The average method was applied to the hclust function in this regard.

Following that, the object generated during this phase was input into the cutreeDynamic function. The cut tree dynamic approach for sample clustering pruning was used with this function.

The method argument was regarded as "tree" in the cutreeDynamic function, and the minimum cluster size was defined as 5. As a result, four clusters were created, one of which contained outlier samples. In DDLPS, just TCGA_MO_A47P was detected as outlier (0 Cluster in Supplementary Fig. 3).

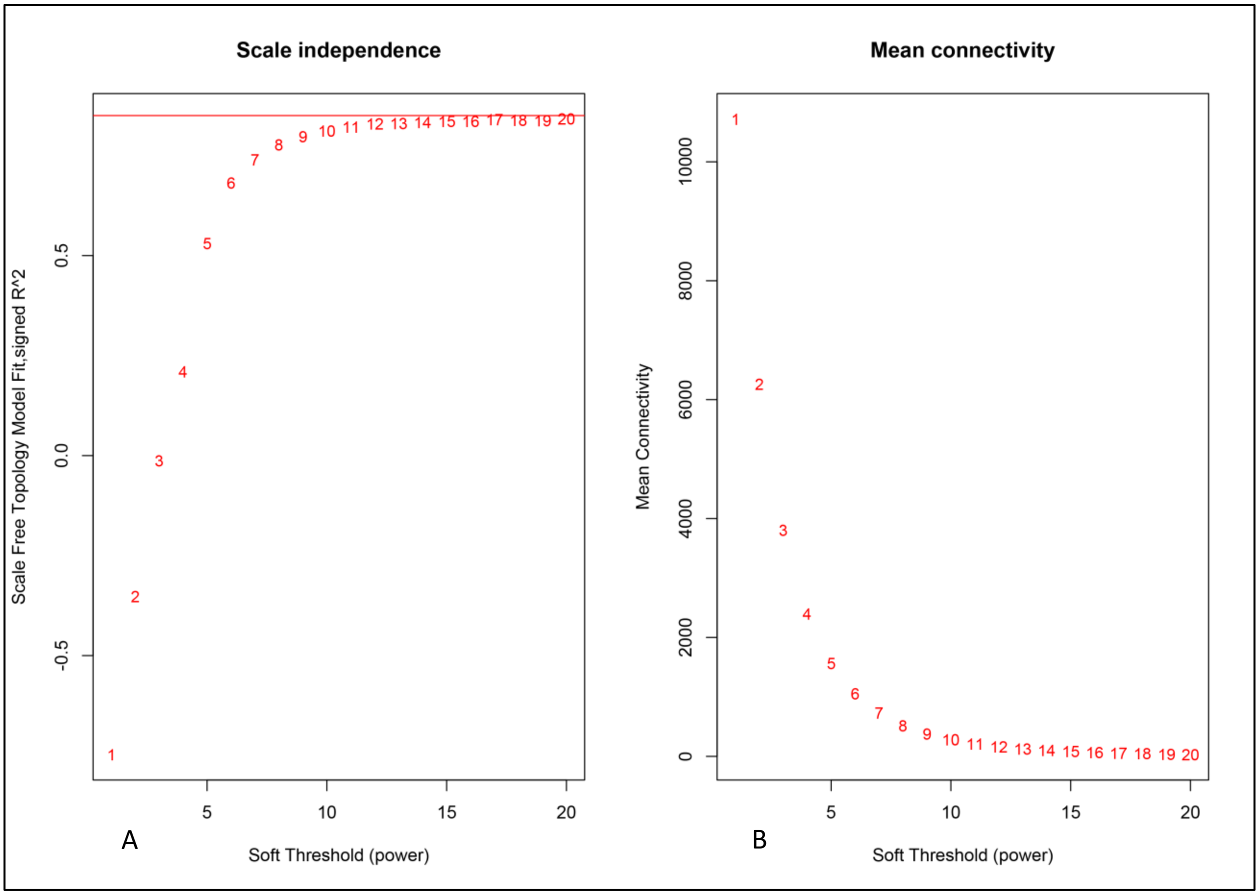

Figure S3: Analysis of network topology for several soft-thresholding powers in WGCNA. (A) Scale-free fit index for different powers (β). (B) Mean connectivity analysis for various soft-thresholding powers (β).

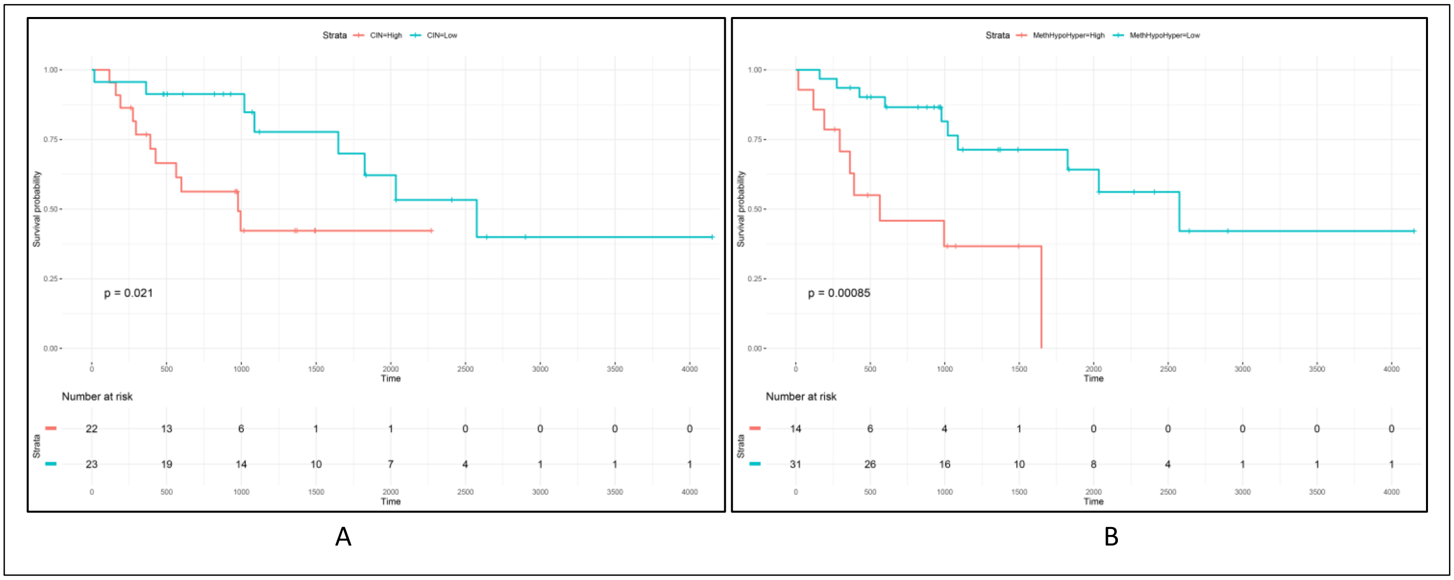

Figure S4: The Kaplan-Meier Plot. (A) KM relates OS for CIN. (B) KM relates OS for Methylation.

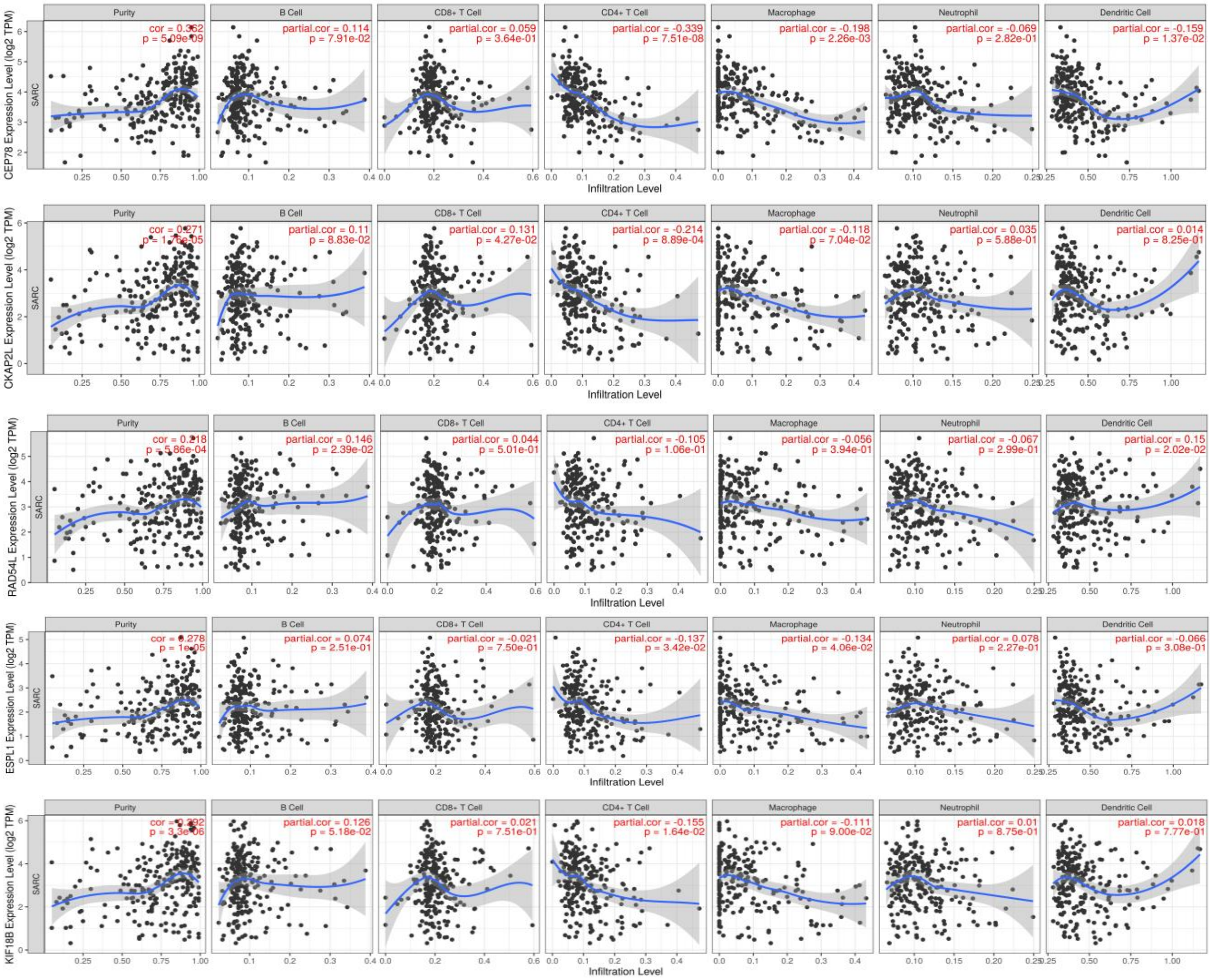

Figure S5: Gene expression correlation with infiltration level curves. Correlation of 5 genes including CEP78, CKAP2L, RAD54L, ESPL1, and KLIF18B expression with tumor purity (left column) and with the infiltration level of immune cell estimated by TIMER (right columns) in sarcoma
